# The association between circulating adiponectin levels, lung function and adiposity in subjects from the general population; data from the Akershus Sleep Apnea Project

**DOI:** 10.1186/s12890-018-0618-4

**Published:** 2018-04-02

**Authors:** Nina F. Caspersen, Helge Røsjø, Allan Flyvbjerg, Mette Bjerre, Anna Randby, Harald Hrubos-Strøm, Torbjørn Omland, Gunnar Einvik

**Affiliations:** 1 Division of Medicine, Akershus University Hospital, Lørenskog, Norway; 2 Institute of Clinical Medicine, University of Oslo, Oslo, Norway; 3 Steno Diabetes Center Copenhagen (SDCC), the Capital Region of Denmark and University of Copenhagen, Copenhagen, Denmark; 4 Medical Research Laboratory, Department of Clinical Medicine, Aarhus University, Aarhus, Denmark; 5 Department of Otorhinolaryngology, Division of Surgery, Akershus University Hospital, Lørenskog, Norway

**Keywords:** Adiponectin, Chronic obstructive pulmonary disease, Adiposity, Lung function

## Abstract

**Background:**

Circulating adiponectin (ADPN) levels are inversely associated with disease severity in patients with chronic obstructive pulmonary disease (COPD), while studies assessing the relationship between ADPN and lung function in subjects from the general population have shown diverging results. Accordingly, we hypothesized that ADPN would be associated with lung function in a population-based sample and tested how abdominal adiposity, metabolic syndrome, and systemic inflammation influenced this association.

**Methods:**

We measured total ADPN in serum, forced vital capacity (FVC) and forced expiratory volume during the 1st second (FEV_1_) in 529 participants (median 50 years, 54.6% males) recruited from the general population. We assessed the association between ADPN and lung function by multivariate linear regression analyses and adjusted for age, gender, height, smoking habits, weight, body mass index, waist-hip ratio, metabolic syndrome, obstructive sleep apnoea (OSA) and C-reactive protein.

**Results:**

The median (interquartile range) level of serum ADPN was 7.6 (5.4–10.4) mg/L. ADPN levels were positively associated with FVC % of predicted (beta 3.4 per SD adiponectin, *p* < 0.001)) in univariate linear regression analysis, but the association was attenuated in multivariate analysis (standardized beta 0.03, *p* = 0.573)). Among co-variates only WHR significantly attenuated the relationship. ADPN levels were also associated with FEV_1_% of predicted in bivariate analysis that adjusted for smoking (beta 1.4, *p* = 0.042)), but this association was attenuated and no longer significant in multivariate analysis (standardized beta -0.06, *p* = 0.254)).

**Conclusion:**

In this population-based sample no association between ADPN and lung function was evident after adjustment for covariates related to adiposity.

**Electronic supplementary material:**

The online version of this article (10.1186/s12890-018-0618-4) contains supplementary material, which is available to authorized users.

## Background

Chronic obstructive pulmonary disease (COPD) is a heterogeneous disease characterized by chronic airway inflammation and reduced lung function [1]. The effects of current treatment options are modest, and COPD is one of the leading causes of morbidity and mortality worldwide [2]. Few serologic biomarkers are currently available for guiding diagnosis, providing prognostic information or evaluating treatment response in COPD [3].

Among proposed serologic biomarkers is adiponectin (ADPN); a protein abundant in serum, which is produced in adipocytes, but also in other tissues including macrophages and airway epithelium [4]. ADPN levels have been found to vary with age, gender, smoking, adiposity and the presence of metabolic syndrome [5–7], some of which may also influence pulmonary function [8]. ADPN is involved in several physiological processes and predominantly exerts anti-inflammatory effects [9, 10]. Previous observations in COPD patients indicate that elevated ADPN levels are associated with accelerated lung function impairment and respiratory mortality [11–13]. In contrast, only limited and contrasting results are currently available regarding the association between ADPN levels and indices of lung function in subjects from the general population [14, 15]. Accordingly, we aimed to assess the association between adiponectin and lung function in a general population sample. We hypothesized that we would find an inverse association between ADPN levels and lung function. We also aimed to assess the impact of adiposity, metabolic syndrome and systemic inflammation on the association between ADPN levels and indices of lung function.

## Methods

### Study design and participants

**Fig. 1:**
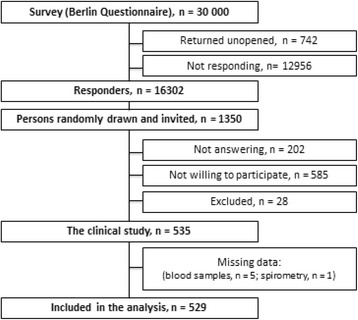
Study flow in the Akershus Sleep Apnea Project

This is a substudy of the Akershus Sleep Apnea Project (ASAP), a two-phased clinical epidemiological study designed to examine sleep apnea in a general population. The recruitment protocol and inclusion/exclusion criteria have previously been reported [16]. In brief, 30,000 subjects between 30 and 65 years, randomly drawn from the Norwegian Personal Registry, received the Berlin Questionnaire by mail in the first phase of the study (Fig. 1). Among the 16,302 respondents, a clinical sample of 535 persons was drawn from pre-specified strata based on equilibrium of age and gender, as well as 2/3 of persons with at least 2 positive risk categories of the Berlin Questionnaire [17].

The study was conducted in accordance with the amended Declaration of Helsinki, and the Regional Committee for Medical Research Ethics in Eastern Norway approved the protocol (1.2005.839). We obtained written informed consent from all participants prior to study commencement.

### Data collection

During an overnight hospital stay, the participants underwent standardized data collection that included medical history, clinical examination, venous blood sampling, polysomnography and spirometry. Tobacco use was categorized as current, former and never. Height, weight, waist and hip circumference were recorded as the mean of three measurements with less than 10% variation, and were used to calculate the body mass index (BMI; weight (kg)/ height [cm]^2^) and the waist-hip-ratio (WHR). Lung function was assessed by Spirosoft spirometry and CardioSoft software (GE HealthCare, Munich, Germany) and recorded as the best performance for forced vital capacity (FVC) and forced expiratory volume during 1st second (FEV_1_) from at least 3 attempts with < 15% variation. FEV_1_ and FVC in percent of predicted [18] were calculated and used as lung function variables in the analyses. Metabolic syndrome was defined according to the National Cholesterol Educational Program criteria [19], based on measurements of systolic blood pressure, total cholesterol, low-density lipoprotein cholesterol, glucose and WHR. Polysomnography was performed and analyzed by two US board-certified polysomnography technicians (Somnologica 3.2 software package (Flaga-Medcare, Buffalo, NY, USA)). Obstructive sleep apnoea (OSA) was defined as an apnoea-hypopnoea index (AHI) equal to or higher than five.

### Biochemical analysis

Venous blood samples were collected in the fasting state in the morning, immediately prepared by centrifugation and separation into plasma and serum samples that were frozen at − 20 °C for a maximum of 1 week before stored at − 80 °C pending analyses, as previously reported [20]. Serum ADPN was determined by a validated in-house time-resolved immunofluorometric assay as previously described [21]. All samples were analyzed in duplicate, with a detection limit of 1.5 μg/L and intra- and interassay coefficients of variation < 5% and < 7%, respectively. C-reactive protein (CRP) was analyzed by a highly sensitive latex immunoassay-kit on a COBAS INTEGRA400 (Roche), with a lower detection limit of 0.1 mg/L.

### Statistical analysis

Data are presented as mean ± standard deviation (SD) or median (quartile [Q] 1–3) for data with a non-normal distribution. We grouped the participants into tertiles of serum ADPN levels and used one-way analysis of variance, Kruskal-Wallis test or chi-square tests to compare clinical data between the groups. We applied multivariate linear regression to explore the association between ADPN and chosen covariates. Linear regression was also used to examine the univariate association between lung function and ADPN, as well as the covariates smoking status, weight, BMI, WHR, metabolic syndrome and CRP. We used FEV_1_ and FVC in percent of predicted as dependent variables, thus, additional adjustment for the covariates age, gender, ethnicity and height were not considered. The multivariate analyses were performed in two stages; we first added adiposity variables, metabolic syndrome and CRP one-by-one to separately evaluate the impact of these features on the association between ADPN and lung function. In the final multivariate linear regression analysis all covariates were first entered simultaneously followed by a stepwise removal of the covariate with the highest *p*-value until all covariates were significantly associated with lung function. Adiponectin was kept in all steps independently of p-value. All regression models were assessed for collinearity. The statistical analyses were performed with Statistical Package for Social Sciences 22, and a *p*-value < 0.05 was regarded as statistical significant.

## Results

The median (Q 1-3) ADPN level in the total population was 7.6 (5.4–10.4) mg/L. Indices of lung function, demographic and clinical characteristics, and laboratory data of the participants are presented stratified according to tertiles of serum ADPN concentrations in Table 1.

**Table 1:** Characteristics of the participants according to tertiles of serum adiponectin levels

		ADPN tertiles			
Variables	Total	Tertile 1	Tertile 2	Tertile 3	*p*
	*n* = 529	*n* = 176	*n* = 176	*n* = 177	
ADPN, mg/L^d^	7.6 (5.4, 10,4)	4.6 (3.8, 5.4)	7.6 (6.7, 8.3)	12.6 (10.3, 15.4)	N/A
Age, years	48.6 (11.3)	46.4 (11.2)	48.6 (10.9)	50.7(11.0)	0.002^a^
Female gender	240 (45.4)	40 (22.7)	74 (42.0)	126 (71.2)	< 0.001^b^
Height, cm	173 (9)	176 (8)	174 (9)	170 (8)	< 0.001^a^
Smoking status					0.860^b^
Current smokers	138 (26.8)	45 (25.9)	50 (29.1)	43 (25.4)	
Previous smokers	184 (35.7)	64 (36.8)	62 (36.0)	58 (31.5)	
Never smokers	193 (37.5)	65 (37.4)	60 (34.9)	68 (40.2)	
Weight, kg	86.7 (17.3)	94.2 (15.4)	88.3 (17.2)	77.6 (15.2)	< 0.001^a^
BMI, kg/m^2^	28.9 (4.98)	30.5 (4.6)	29.2 (5.0)	26.9 (4.6)	< 0.001^a^
WHR	0.94 (0.1)	0.99 (0.08)	0.95 (0.09)	0.88 (0.09)	< 0.001^a^
Metabolic syndrome	174 (32.9)	89 (50.6)	58 (33.0)	27 (15.5)	< 0.001^b^
OSA	293 (55.6)	71 (30.3)	75 (32.1)	88 (37.6)	0.167^b^
CRP, mg/L^d^	1.26 (0.65, 2.94)	1.48 (0.78, 3.20)	1.44 (0.69, 3.28)	0.92 (0.50, 2.09)	0.001^c^
FEV_1_	3.41 (0.88)	3.64 (0.89)	3.51 (0.93)	3.09 (0.77)	< 0.001^c^
FVC	4.15 (1.03)	4.37 (0.99)	4.27 (1.08)	3.81 (0.93)	< 0.001^c^
FEV_1_, % of predicted	103.25 (16.11)	101.19 (16.11)	104.45 (16.66)	104.09 (15.44)	0.115^a^
FVC, % of predicted	103.96 (16.33)	99.52 (15.78)	104.79 (16.22)	107.53 (16.05)	< 0.001^a^

*ADPN* adiponectin, *FEV*_*1*_ forced expiratory volume during 1st second, *FVC* forced vital capacity, *BMI* body mass index, *WHR* waist-hip ratio, *OSA* obstructive sleep apnoea, *CRP* C-reactive protein

^a^analyses of variance, ^b^chi-square tests, ^c^Kruskal-Wallis

Continuous variables are expressed as mean (standard deviation) or ^d^median (interquartile range) and categorical variables are expressed as number (%). Metabolic syndrome is defined according to the National Cholesterol Education Program criteria. OSA, Obstructive Sleep apnoea is defined as apnoea-hypopnoea index ≥5

**Fig. 2:**
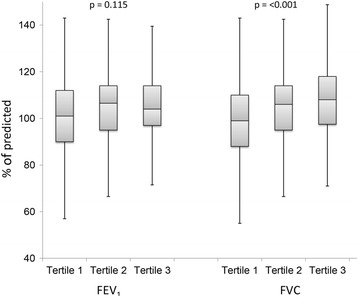
Lung function in % of predicted according to tertiles of adiponectin

The absolute values of FEV_1_ and FVC decreased by increasing ADPN tertiles. For FEV_1_% of predicted, there was no significant difference between ADPN tertiles, while FVC% of predicted increased by increasing ADPN levels (Fig. 2). Age and proportion of females increased across tertiles of serum ADPN, while height, weight, BMI, WHR, CRP and the proportion of participants with metabolic syndrome decreased. Smoking status and prevalence of OSA did not differ significantly between the groups. Univariate and multivariate analysis of the association between ADPN and selected covariates are shown in Additional file 1: Table S1. All variables except smoking were significantly correlated with ADPN in univariate analysis, while age, gender, WHR and metabolic syndrome remained significantly associated with ADPN in multivariate analyses.

The crude associations between FEV_1_ or FVC % of predicted and ADPN, smoking status, anthropometric parameters, metabolic syndrome, OSA and CRP are shown in Table 2.

**Table 2:** Univariate associations between lung function expressed as FEV_1_ and FVC in percent of predicted, adiponectin, smoking status, indices of adiposity, metabolic syndrome, obstructive sleep apnoea and C-reactive protein

	FEV_1_% predicted		FVC % predicted	
	Unstandardized beta (CI)	*p*	Unstandardized beta (CI)	*p*
ADPN (per SD)	1.012 (− 0.365, 2.388)	0.149	3.414 (2.047, 4.781)	< 0.001
Current smoking	−5.444 (−8.483, − 2.406)	< 0.001	−2.219 (−5.410, 0.972)	0.173
Previous smoking	0.645 (− 2.197, 3.487)	0.656	− 1.143 (−4.096, 1.811)	0.448
Weight (per 5 kg)	−0.661 (− 1.055, − 0.268)	0.001	− 1.119 (− 1.510, − 0.728)	< 0.001
BMI kg/m^2^	−0.466 (− 0.740, − 0.192)	0.001	−0.647 (− 0.922, − 0.371)	< 0.001
WHR	−38.297 (− 52.041, − 24.553)	< 0.001	−62.199 (−75.488, − 48.909)	< 0.001
Metabolic syndrome	−4.905 (−7.808, − 2.001)	0.001	− 7.375 -10.289, − 4.471)	< 0.001
OSA	−2.143 (− 4.911, 0.625)	0.129	− 3.923 (− 6.725, − 1.120)	0.006
CRP (per SD)	− 2.157 (− 3.524, − 0.791)	0.002	− 2.071 (− 3.457, − 0.685)	0.003

*FEV*_*1*_ forced expiratory volume during 1st second, *FVC* forced vital capacity, *CI* confidence interval, *ADPN* adiponectin, *SD* standard deviation, *BMI* body mass index, *WHR* waist-hip ratio, *OSA* obstructive sleep apnoea, *CRP* C-reactive protein

There was no significant association between FEV_1_% of predicted and ADPN. A positive association with ADPN was found after adjustment for smoking, while the models including each of the other co-variates found no associations between FEV_1_% of predicted and ADPN. In contrast, FVC% of predicted was significantly associated with ADPN, measures of adiposity, metabolic syndrome, OSA and CRP, but not with smoking. The association between ADPN and FVC in % of predicted remained positive after adjusting for smoking status, weight, BMI, metabolic syndrome, OSA and CRP one by one in separate linear regression models. However, the association was attenuated and no longer significant when adjusting for WHR (Table 3).

**Table 3:** The associations between adiponectin (ADPN) and lung function in percent of predicted adjusting for different covariates one by one

		Unstandardized beta (95% CI) ADPN (per SD)			
		FEV1% of predicted	*p*	FVC % of predicted	*p*
Unadjusted		1.012 (− 0.365, 2.388)	0.149	3.414 (2.047, 4.781)	< 0.001
Adjusted for	Weight (per 5 kg)	0.060 (− 1.445, 1.566)	0.937	2.166 (0.681, 3.651)	0.004
	BMI	0.303 (− 1.139, 1.744)	0.680	2.658 (1.229, 4.087)	< 0.001
	WHR	− 1.213 (− 2.771, 0.345)	0.127	0.425 (− 1.085,1.935)	0.581
	Current smoking	1.393 (0.048, 2.739)	0.042	3.556 (2.172, 4.941)	< 0.001
	Previous smoking	1.380 (0.017, 2.742)	0.047	3.529 (2.141, 4.917)	< 0.001
	Metabolic syndrome	0.353 (−1.078, 1.785)	0.628	2.610 (1.195, 4.025)	< 0.001
	OSA	1.090 (−0.299, 2.480)	0.124	3.282 (1.90, 4.664)	< 0.001
	CRP(per SD)	0.834 (−0.537, 2.206)	0.232	3.262 (1.897, 4.626)	< 0.001

*FEV*_*1*_ forced expiratory volume during 1st second, *FVC* forced vital capacity, *CI* confidence interval, *ADPN* adiponectin, *BMI* body mass index, *WHR* waist-hip ratio, *OSA* obstructive sleep apnoea, *CRP* c-reactive protein

In the final multivariate models that included smoking status, BMI, WHR, metabolic syndrome, OSA and CRP, ADPN was not associated with any of the lung function variables (Table 4).

**Table 4:** The association between lung function and serum adiponectin levels adjusted for multiple covariates

	FEV1% of predicted	*p*	FVC % of predicted	*p*
	Standardized B (CI)		Standardized B (CI)	
Adiponectin/SD	−0.056 (−2.390, 0.632)	0.254	0.027 (−1.083, 1.956)	0.573
WHR	−0.264 (−58.269, −27.208)	< 0.001	− 0.360 (−76.027, −44.799)	< 0.001
Current smoking	− 0.170 (−8.940, −3.075)	< 0.001	−0.083 (−6.010, − 0.113)	0.042
CRP/SD	− 0.125 (− 3.235, − 0.660)	0.003	−0.103 (− 2.957, − 0.368)	0.012

*FEV*_*1*_ forced expiratory volume during 1st second, *FVC* forced vital capacity, *CI* confidence interval, *ADPN* adiponectin, *SD* standard deviation, *WHR* waist-hip ratio, *CRP* c-reactive protein

## Discussion

In the present population-based sample, we found that ADPN was positively associated with lung function after adjustment for age, gender, height and smoking, but this association was attenuated and not significant when adjusting also for indices of adiposity, metabolic syndrome, CRP and OSA. This result reflects the negative impact of adiposity on both lung function and serum ADPN levels.

The negative association between ADPN and severity of COPD previously observed in clinical cohorts has no established pathophysiological explanation, but could imply that ADPN is linked to disease development and progression. In the present study, we investigated whether a negative association between ADPN and lung function was present in a Caucasian general-population sample, hypothetically reflecting ADPN as an early marker of obstructive airway limitation. The two previous population-based studies assessing the relationship between ADPN and lung function have reported diverging results. A Japanese study reported a negative association between ADPN and FEV_1_/FVC in unadjusted analyses, as well as accelerated decline in FEV_1_ with higher ADPN levels after adjustment for age, gender, height, smoking and BMI. In contrast, the CARDIA study, like the present study, observed an inverse association between ADPN and lung function in unadjusted analyses, but this association was attenuated and no longer significant in analysis that adjusted also for insulin resistance and systemic inflammation [14]. Thus, we find it plausible that circulating ADPN levels are primarily associated with indices of adiposity, and that univariate associations between ADPN levels and pulmonary function reflect an inverse association between adiposity and pulmonary function.

A novel finding in the present study is the important role of WHR as a confounder in the association between ADPN and lung function. While previous studies and the present study found that BMI alone did not attenuate the association between ADPN and lung function, WHR alone attenuated the association between ADPN and FVC in the present study. WHR also remained significantly associated with both FEV_1_% of predicted and FVC % of predicted in multivariate analysis. Abdominal adiposity is a surrogate marker of visceral adiposity and is associated with serum levels of adiponectin, metabolic disease and systemic inflammation, but also results in mechanical restriction that may affect lung function and FVC in particular. The results from the present study indicate that the role of mechanic restriction from abdominal obesity contributes more to lung function than the changes in ADPN secretion and metabolic disease associated with this condition,

Interestingly, there were differences between FEV_1_ and FVC in % of predicted as dependent variables. While FVC is a measure of lung volume, FEV_1_ is also an indicator of airway obstruction. In our study, ADPN was only associated with FVC % of predicted in univariate analyses. Also, while all types of adiposity-related factors further attenuated the association between ADPN and FEV_1_% of predicted, only WHR attenuated the positive association between FVC % of expected and ADPN. This might reflect that CRP and metabolic syndrome, as indicators of inflammatory and metabolic consequences of adiposity, are more important for FEV_1_ than for FVC. This is in accordance with results in the CARDIA-study, which found metabolic syndrome and insulin resistance as well as systemic inflammation to attenuate the relationship between FEV_1_ and adiponectin. These factors will be important to include in multivariate analysis when evaluating the relationship between ADPN and lung function.

There are several differences in the three presented population-based studies, besides the choice of covariates, which could explain the discrepant conclusions. One factor that could impact the result is ethnic variations with our study including primarily Caucasian subjects, the CARDIA study including a mixed American population while the third study examined a Japanese population. The CARDIA study also included younger individuals (mean age 40 years), the present study subjects with mean age of 50 years, and the Japanese study subjects with mean age of ~ 60 years. As ADPN levels are known to increase with age [22], different age groups could potentially also contribute to the diverging results between these three general population-based cohorts.

A limitation compared to previous studies is the cross-sectional design that makes us unable to evaluate changes in lung function and causal pathways. On the other hand, compared to CARDIA, we were able to analyze ADPN samples obtained at the same time point as the spirometry. The ASAP cohort consisted of individuals with more risk factors for obstructive sleep apnoea than a strict general population sample and were as a group more obese, more sleepy and had a higher rate of diabetes and prior myocardial infarction than the general population sample responding to the Berlin questionnaire in the first part of the study. OSA is associated with lower ADPN levels and is a possible confounder in the examined relationship between ADPN and lung function. However, the prevalence of OSA did not differ between the tertiles of ADPN and the association between FVC% of predicted and ADPN remained significant after adjustment for OSA and metabolic syndrome. Also mean ADPN levels and measures of lung function in the study sample were comparable to those observed in previous general population studies [15, 23]. Based on this we believe our results are valid also for subjects from the general population. Finally, only total ADPN levels were measured and therefore information regarding different isoforms is not available.

## Conclusions

In this population-based study, we found a positive association between ADPN and FEV_1_ and FVC after adjustment for age, gender, height and smoking that was attenuated after additional adjustment for indices of abdominal adiposity. Accordingly, future assessment of the association between ADPN and lung function should take both multiple indices of adiposity and its metabolic consequences into consideration.

## Additional files

Additional file 1: **Supplementary table 1.** Univariate and multivariate adjusted associations between ln Adiponectin and chosen covariates. (DOCX 15 kb)

Additional file 2: Dataset. (XLSX 59 kb)

## Abbreviations

ADPN: Adiponectin
ASAP: Akershus Sleep Apnea Project
BMI: Body mass index
CARDIA: Coronary artery risk development in young adults
COPD: Chronic obstructive pulmonary disease
CRP: C-reactive protein
FEV_1_: Forced expiratory volume in 1 second
FVC: Forced vital capacity
SD: Standard deviation
WHR: Waist-hip ratio
