# Supplementary material for: The association between circulating adiponectin levels, lung function and adiposity in subjects from the general population; data from the Akershus Sleep Apnea Project
Source: BMC Pulm Med. 2018 Apr 2;18:54. doi: 10.1186/s12890-018-0618-4 (PMC5879541; doi:10.1186/s12890-018-0618-4)
Supplement: Supplementary file 1 — Supplementary table 1. Univariate and multivariate adjusted associations between ln Adiponectin and chosen covariates. (DOCX 15 kb) [file 12890_2018_618_MOESM1_ESM.docx]

**Table S1.** Univariate and multivariate adjusted associations between ln Adiponectin and chosen covariates.

|  | **Univariate model** | | **Multivariate model** | |
| --- | --- | --- | --- | --- |
|  | Unstandardized β | p | Standardized β | p |
| Age per 5 years | 0.041 (0.022, 0.059) | <0.001 | 0.279 (0.045, 0.080) | <0.001 |
| Gender | -0.441 (-0.519, -0.363) | <0.001 | -0.230 (-0.359, -0.106) | <0.001 |
| Height per 5 cm | -0.088 (-0.112, -0.065) | <0.001 | -0.053 (-0.145, 0.114) | 0.818 |
| Smoking status |  |  |  |  |
| Current | 0.021 (-0.077, 0.120) | 0.668 | 0.024 (-0.062, 0.116) | 0.549 |
| Previous | -0.032 (-0.123, 0.059) | 0.488 | 0.043 (-0.039, 0.129) | 0.290 |
| Never | 0.014 (-0.077, 0.104) | 0.769 |  |  |
| Weight per 5 kg | -0.062 (-0.073, -0.050) | <0.001 | 0.224 (-0.093, 0.158) | 0.612 |
| BMI, kg/m2 | -0.032 (-0.040, -0.024) | <0.001 | -0.255 (-0.099, 0.048) | 0.491 |
| WHR | -2.685 (-3.063, -2.307) | <0.001 | -0.371 (-2.554, -1.292) | <0.001 |
| Metabolic syndrome | -0.345 (-0.432, -0.258) | <0.001 | -0.153 (-0.251, -0.077) | <0.001 |
| OSA | -0.110 (-0.196, -0.024) | 0.013 | 0.020 (-0.059, 0.100) | 0.617 |
| Ln CRP | -0.067 (-0.106, -0.028) | 0.001 | -0.004 (-0.038, 0.035) | 0.927 |
